# The Present and Future Applications of Technology in Adapting Medical Education Amidst the COVID-19 Pandemic

**DOI:** 10.2196/20190

**Published:** 2020-07-17

**Authors:** Ridhaa Remtulla

**Affiliations:** 1 Birmingham Medical School College of Medical and Dental Sciences University of Birmingham Birmingham United Kingdom

**Keywords:** medical education, technology, coronavirus, medical students, COVID-19, pandemic, online lecture, virtual reality

## Abstract

The coronavirus disease (COVID-19) pandemic has not only been catastrophic toward patient health but has also proven to be incredibly disruptive to several industries and sectors, including medical education. However, many medical schools have employed various technological solutions in order to minimize the disruption to medical education during this unpredictable time. This viewpoint reviews the various current and potential applications of technology in order to adapt medical education amidst a global pandemic.

## Introduction

The global emergency of coronavirus disease (COVID-19) has been exceptionally disruptive for several industries and sectors, including medical education. Abrupt university closures across the world have posed significant challenges for medical schools on an international scale, with a considerable number of universities graduating their final year medical students in haste in order to contribute to national workforces, whilst other more junior medical students have faced premature ends to their academic year [1].

Despite the unexpected imposition of lockdowns, universities were able to leverage technological solutions to ensure continuity of their courses. Nevertheless, more difficult decisions lie ahead for medical schools. Educational institutions were nearing the end of the academic year when countries entered lockdown phases. Thus, establishments must consider how medical education, both preclinical and clinical, will be delivered with the commencement of the new academic year.

This paper aims to identify the various technological solutions that are allowing for the adaptation of medical education in these unpredictable times, and how more novel digital solutions may be used in the future to enable students to seamlessly progress through medical school in the age of the COVID-19 pandemic.

## Online Lectures

Following national lockdowns, numerous universities rapidly switched to delivering live or prerecorded online lectures. Third-party organizations have also been holding online lectures and podcast series to help ensure medical students continue to receive their education. This is not a novel application of technology, as medical schools frequently record their lectures to allow students to access learning materials at later points. Online lectures are a highly familiar method of teaching, and multimedia resources are regularly used by medical students to supplement their learning. Indeed, studies have demonstrated that online lectures can increase the speed of knowledge acquisition, allow students to manage stress better, and most importantly, they can improve learning outcomes from the course when compared to in-person lectures [2,3].

One study of 222 medical and dental students found that 66% of students between years 2 and 4 felt physical lectures should not be a compulsory component of their medical education, highlighting newer student preferences and raising the argument that traditional lectures may indeed be an outdated method of teaching [4]. It should be considered that based on this data, students may be preferring the current norm of online lectures to conventional lectures. Online lectures are also exceptionally customizable to a student’s specific learning needs, for example, by allowing individuals to increase or reduce the speed of the lecture to suit their personal pace. Considering this in combination with the fact that this educational medium has been shown to result in better outcomes for students, perhaps online lectures will become a permanent alteration in many medical courses.

## Interactive Technologies to Replace Hands-on Learning

Digital tools for learning practical medicine have been used for some time now, a prime example being the Anatomage table—a large interactive screen that students can use to virtually dissect the human body and observe its structures [5]. Similar software programs have also emerged that allow students to access these facilities in a portable manner (eg, mobile or laptop applications that enable individuals to study anatomy via 3D computer models). These portable solutions could act as viable replacements for the dissection and prosection aspects of most medical programs.

In order to allow for the increased demand that COVID-19 will bring, many hospitals postponed elective surgeries, and the impact of this on training surgeons must be considered. Alternatives such as Touch Surgery, an innovative surgical simulation app, may be used by trainee surgeons in order to maintain their practice [6]. In recent years, some surgeons have also chosen to live stream their operations, and this has been achieved through various modalities. Live streaming of surgeries with a camera alone can be thought of as cutting-edge, but some surgeons have employed even more novel methods (eg, streaming surgeries through the use of wearable devices such as Snapchat glasses [7]). Live streaming surgeries through any viable medium has tremendous utility in the age of COVID-19, since it allows for the continuity of medical education in a time where being physically present in the operating room may not be possible. The fact that there has been a relatively extensive practice of live streaming in the past suggests that this technology may well become the status quo in the case of canceled clinical placements or clerkships.

## Online Examinations

For institutions that continued to hold final year examinations for their students, remote online examination systems were used, demonstrating that remote testing can be employed in case of future disruption. The Situational Judgement Test (SJT) is a national exam sat by all British medical students in their final year. This had been switched from paper to online examinations for the upcoming academic year, prior to the outbreak of COVID-19. Students can choose to take the SJT as an online examination at either a local testing center or from their own home computer; this highlights that it is certainly possible to hold remote, online examinations in case restrictive social distancing measures are in place during the exam period.

Another potential solution may be to conduct remote, online open book examinations (OBEs). As a result of the COVID-19 outbreak, Imperial College London held high-stakes, final year medical examinations in this manner. Interestingly, the median mark for the OBEs were equivalent to the median marks of the previous 3 years prior to the COVID-19 pandemic [8]. Due to the nature of the questions, which mainly focused on applying knowledge as opposed to basic recall, students were not provided an unfair advantage by making the examination open book [8].

Alternatively, if institutions wish to conform to the traditional closed-book examinations, there are technologies available in order to maintain appropriate levels of invigilation. All medical schools have a zero-tolerance policy to inappropriate academic conduct, necessitating the use of precautionary measures to prevent unethical behavior concerning examinations. This may be tackled through the use of technologies that track eye movements, keystrokes, and background noises to recognize potential cheating behavior [9].

## Telemedicine

Telemedicine, which is the use of technology in order to deliver health care remotely, is becoming increasingly important in medical service delivery. However, its applications could also be extended to medical education, and the current COVID-19 crisis is evidence of this. Doctors have resorted to simple yet effective “webcams on wheels,” enabling them to conduct virtual ward rounds remotely whilst a staff member who is physically present at the hospital maneuvers a computer with a camera around the ward using a trolley [10]. By allowing groups of medical students to connect to similar devices, for example, through a video conference call, students would be able to continue participating in ward rounds as they usually do during their clinical clerkships.

Additionally, a pilot study investigated the use of telemedicine technology toward building a formative and remote objective structured clinical examination (OSCE) [11]. The “teleOSCE” involved medical students speaking to a patient actor over video conferencing. This was found to be economically feasible and was positively received by participating students [11]. Thus, an additional use of telemedicine could also be for summative practical examinations, which would otherwise require canceling.

## Virtual Reality

Although the use of virtual reality (VR) is not as widespread when compared to other devices such as touchscreen tablets or smartphones, the challenges posed by COVID-19 could potentially be a turning point for this technology. Medical schools may be forced to cancel clinical placements in the coming months to protect their students from undue exposure to COVID-19. Through the use of VR, there is potential to digitally reconstruct aspects of the clinical environment and simulate clinical learning. Oxford Medical Simulation is a company offering a VR medical education platform where students are able to examine, diagnose, treat, and take histories from digitally simulated patients within a virtual clinical environment [12]. These interactive and immersive scenarios will adapt based on the actions of the student, thus closely mimicking what students would learn in real life. One scoping review identified 21 papers in which VR had been used in medical training. Of these 21 papers, 74% found improved learning using VR. Doctors who were trained through VR were also reported to have a higher level of accuracy in their medical practice, indicating that this is an effective method for teaching in a medical context [13].

With the vast majority of clinical schools canceling OSCEs for the 2019/20 academic year due to COVID-19, perhaps VR technology also acts as a more novel solution to ensure the continuation of practical examinations. Companies such as Medical Realities do offer VR OSCE practice sessions, demonstrating that this is certainly within the grasp of current technological capabilities [14]. In combination with the aforementioned telemedical solutions, recreating a realistic summative OSCE that tests both practical skills and communication abilities is very possible.

## Key Challenges

It is clear that the golden age of technology aids in minimizing the disruption to medical education in these current times, with online resources and lectures playing a key role in the current continuation of medical education. However, it is crucial to identify those who may fall through the cracks during this period of time, especially individuals who may have difficulty accessing high-speed internet—for example, those training in less developed parts of the world. Additionally, some of the technologies discussed—particularly VR—are costly and may not be economically feasible at their present prices. Microsoft HoloLens, for example, costs $3500 per device [15].

## Conclusion

There is no question that the coming academic year will be a challenge for both medical students and medical schools. However, health care professionals have always been recognized and respected for their resilience, and universities are already using technology to ensure the seamless progression of their students through medical school. If nothing else, the adaptations toward remote education solutions in the era of COVID-19 may lead to further innovation in the field, and a potential revolution in the way medical education is delivered through novel technologies.

## Abbreviations

COVID-19: coronavirus disease
OBE: online open book examination
OSCE: objective structured clinical examination
SJT: Situational Judgement Test
VR: virtual reality
